# From Years of Pain to a Name: Fibromyalgia Uncovered

**DOI:** 10.7759/cureus.98981

**Published:** 2025-12-11

**Authors:** Sara Ali, Archana Banda, Carlos Ramirez-Matos, Peter Cohen

**Affiliations:** 1 Medicine, Nova Southeastern University Dr. Kiran C. Patel College of Osteopathic Medicine, Clearwater, USA; 2 Family Medicine, Palmetto General Hospital, Hialeah, USA; 3 Family Medicine, Nova Southeastern University Dr. Kiran C. Patel College of Osteopathic Medicine, Fort Lauderdale, USA

**Keywords:** chronic disease, chronic widespread pain, fibromyalgia, multisystem condition, pain management

## Abstract

Fibromyalgia is a chronic multisystem disorder marked by widespread pain, fatigue, and sleep and mood disturbances, predominantly affecting women. Its multifactorial etiology and diagnosis by exclusion often delay recognition. A multimodal approach, with reassurance, shared treatment goals, education, cognitive behavioral therapy (CBT), and exercise, is critical. Effective pharmacologic options include duloxetine, milnacipran, pregabalin, and amitriptyline. This report describes a female in her late thirties who presented to the clinic with chronic widespread pain. Her past medical history was significant for a motor-vehicle accident four months ago, bipolar II disorder, major depressive disorder, post-traumatic stress disorder, irritable bowel syndrome, cardiomyopathy, stage 3 chronic kidney disease, hidradenitis suppurativa, and melanoma. When repeated laboratory tests and imaging failed to provide answers, it became increasingly clear that this patient's case was particularly challenging to diagnose, especially due to existing comorbidities. However, after ruling out the common causes for diffuse chronic pain, fibromyalgia was diagnosed. This case illuminates the importance of clinicians being patient advocates throughout the journey and listening attentively to patients' concerns even when laboratory results and imaging appear unremarkable.

## Introduction

Fibromyalgia is a multisystemic condition that is known to cause chronic widespread pain, fatigue, disturbances in sleep and mood, along with additional functional impairments. This is a commonly unrecognized syndrome that predominantly affects women and impacts approximately 2%-5% of the adult population in the United States [1]. This condition primarily impacts the musculoskeletal system. However, it is also known to affect the gastrointestinal, genitourinary, and nervous systems. Overall, it is important to be able to rule out other conditions that present similarly to fibromyalgia. For instance, rheumatic diseases, including rheumatoid arthritis, systemic lupus erythematosus, inflammatory arthritis, and polymyalgia rheumatica, can also present with chronic widespread pain; however, these disorders are generally distinguished by their inflammatory nature [2]. Additional differential diagnoses include hypothyroidism, hyperparathyroidism, and vitamin D deficiency, which may cause musculoskeletal pain and fatigue but are typically accompanied by abnormal hormonal or vitamin levels. Furthermore, adverse effects from medications, such as statins, chemotherapeutic agents, and bisphosphonates, can also contribute to widespread musculoskeletal pain [3].

The etiology of this condition remains multifactorial, including genetics and emotional-cognitive factors, necessitating a holistic multimodal treatment approach [4]. There tends to be a trigger, such as physical or emotional trauma, in some patients [5]. However, since this rare condition can only be diagnosed by exclusion, establishing the disease often takes several years. In order to aid in the diagnosis of fibromyalgia, multiple diagnostic criteria have been developed over the years. For example, the 2016 American College of Rheumatology (ACR) Fibromyalgia Diagnostic Criteria states that all of the following conditions must be met for a diagnosis: a Widespread Pain Index (WPI) score of ≥7 and a Symptom Severity Scale (SSS) score of ≥5, or a WPI score of 4-6 and an SSS score of ≥9; generalized pain present in at least four of five regions (left upper, right upper, left lower, right lower, and axial); and symptoms that have persisted for at least three months. Moreover, the AAPT (Analgesic, Anesthetic, and Addiction Clinical Trial Translations, Innovations, Opportunities, and Networks-American Pain Society (ACTTION-APS) Pain Taxonomy) 2019 Diagnostic Criteria for Fibromyalgia classifies fibromyalgia as pain in at least six of the nine possible sites (head, left arm, right arm, chest, abdomen, upper back and spine, lower spine including the buttocks, left leg, and right leg), moderate to severe sleep problems or fatigue, and symptoms present for at least three months [1].

Altogether, it is pivotal for physicians to provide reassurance and collaborate with patients in developing shared treatment goals. This can include a personalized plan involving both pharmacological and non-pharmacological management of fibromyalgia. Pharmacological therapies encompass pain modulators such as serotonin and norepinephrine reuptake inhibitors (SNRI), antidepressants, and antiepileptic agents [6]. Effective medications for decreasing diffuse chronic pain include duloxetine, milnacipran, pregabalin, and amitriptyline [7]. Additionally, non-pharmacological management of fibromyalgia involves cognitive behavioral therapy (CBT), aerobic exercise, strength training, aquatic therapy, meditative movement therapies, cryotherapy, acupuncture, and mindfulness-based stress reduction therapies [8]. Given the complicated nature of this condition, a combination of pharmacological treatments, non-pharmacological interventions, and patient education is crucial in managing pain, overall function, and patient quality of life.

## Case presentation

The patient is a Caucasian female in her late thirties who presented to the family medicine clinic for a follow-up appointment regarding her chronic widespread pain. She reported severe joint pain in her wrists, elbows, and hips bilaterally, as well as diffuse abdominal pain. At this visit, the patient mentioned persistent nausea, vomiting, and diarrhea, which began four days prior, resulting in poor oral intake and an inability to tolerate food.

Her past medical history consists of a motor-vehicle accident four months ago, bipolar II disorder, major depressive disorder, post-traumatic stress disorder, irritable bowel syndrome, cardiomyopathy, stage 3 chronic kidney disease, hidradenitis suppurativa, and melanoma. Further, her past surgical history includes a hysterectomy and a gastric sleeve procedure. Current medications include lamotrigine and sertraline for mood stabilization and depression.

The patient recently relocated from another state and reported experiencing heightened stress from her current living situation, as well as poor sleep quality and financial hardships. She has a 15-pack-year smoking history, having smoked three packs of cigarettes daily but quit smoking eight months ago. She denied alcohol and recreational drug use. The patient mentioned decreased activity levels and difficulties performing daily tasks such as brushing her hair and walking to the grocery store. Her diet primarily consists of fast food, and she is currently unemployed and receiving disability insurance. Family history is notable for diabetes mellitus and hypertension on both maternal and paternal sides.

Two months prior, the patient was admitted to the hospital with a chief complaint of “pain all over.” During that admission, a series of lab and imaging tests were performed to identify a diagnosis for her diffuse musculoskeletal pain. Lab studies revealed her erythrocyte sedimentation rate (ESR), C-reactive protein (CRP), vitamin B12, and folate to be established within the typical reference interval. Additionally, the anti-cyclic citrullinated peptide (anti-CCP) and rheumatoid factor tests were negative. A thyroid panel with TSH was also within normal limits. Regarding imaging, CT scans of the cervical spine, thoracic spine, lumbar spine, right hip, and abdomen and pelvis revealed no signs of acute fracture or dislocations. As a result, the patient was prescribed methocarbamol upon discharge and recommended a follow-up with her primary care physician.

**Figure 1:**
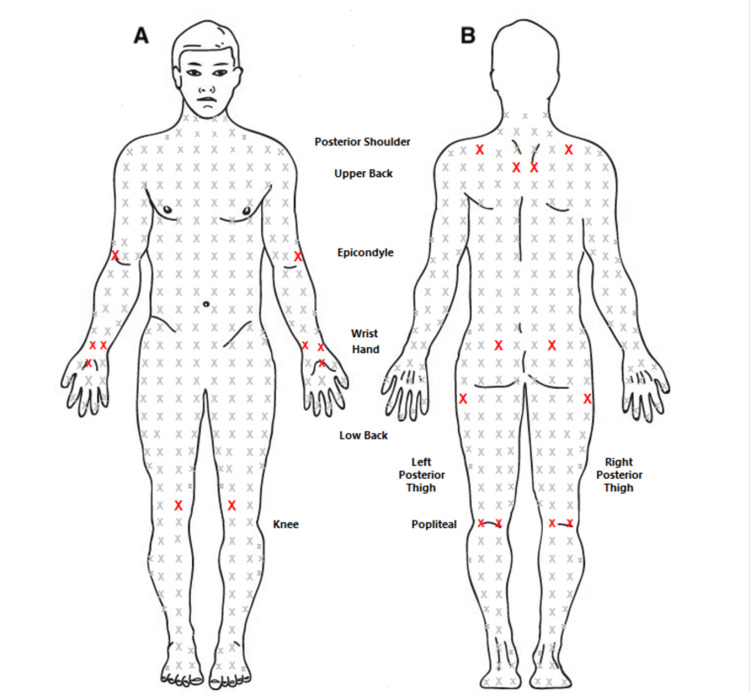
Overview of all identified tender points: Red X marks indicate positive tender point regions Anterior (A) and posterior (B) tender points are shown, with areas of tenderness indicated by red X marks. These marks indicate regions where the patient experienced pain during the tender point assessment, revealing severe bilateral tenderness in the following nine regions: posterior shoulder, upper back, epicondyle, wrist, hand, low back, posterior thigh, popliteal area, and knee. The figure is the original work of the authors.

Upon physical examination at the current clinic visit, the patient appeared fatigued but was alert and oriented to time and place. Inspection of the joints revealed no signs of erythema or swelling, and upon light palpation, the patient expressed hyperacute pain, rated as 10/10 in intensity. Abdominal examination revealed hyperactive bowel sounds. Neurologic examination was unremarkable. In addition, a tender point assessment was performed, which revealed severe bilateral tenderness in the following nine regions: posterior shoulder, upper back, epicondyle, wrist, hand, low back, posterior thigh, popliteal, and knee (as illustrated in Figure 1).

Given that the patient’s symptoms were not supported by abnormal laboratory or imaging findings, a diagnosis of fibromyalgia was made. This diagnosis was supported by the presence of chronic widespread pain for more than three months without another identifiable cause, as well as associated fatigue, sleep disturbance, and comorbid irritable bowel syndrome, a condition frequently associated with fibromyalgia [9]. The patient was referred to a pain medicine specialist the same day and was prescribed pregabalin and tramadol for pain management. She was further counseled on the importance of improving sleep quality and reducing stress.

## Discussion

Overall, this case represents the diagnostic challenges of fibromyalgia in a patient with multiple comorbidities, including bipolar II disorder, major depressive disorder, post-traumatic stress disorder, irritable bowel syndrome, cardiomyopathy, stage 3 chronic kidney disease, hidradenitis suppurativa, and melanoma. The concurrence of these conditions complicated the establishment of a proper diagnosis, as each can contribute to chronic pain, fatigue, or psychosomatic symptoms. Further, the socioeconomic challenges, including financial difficulties, unemployment, and limited access to quality healthcare, likely contributed to the patient’s symptoms and delayed her ability to seek medical treatment.

Given the patient's use of sertraline for depression, the addition of a SNRI such as duloxetine raised concern for potential serotonin syndrome. Thus, tramadol and pregabalin were prescribed for pain management instead. Pregabalin acts by inhibiting the release of excitatory neurotransmitters, while tramadol serves as a centrally acting opioid analgesic. Optimizing lifestyle factors, notably improving sleep quality and reducing stress, are also essential components in the management of fibromyalgia.

Currently, no diagnostic test is available that can be used to confirm fibromyalgia. Rather, diagnosis is based on clinical judgment and the presence of chronic widespread pain for at least three months, along with fatigue and poor sleep, in the absence of another explanatory disease [10]. The WPI and SSS are both questionnaires that aid in assessing the degree and intensity of chronic widespread pain when diagnosing fibromyalgia and are components of the AAPT 2019 Diagnostic Criteria of Fibromyalgia [1]. Thus, it is imperative to keep fibromyalgia on the list of differentials since it is considered a diagnosis of exclusion.

In terms of pain management, pharmacological therapy aims to provide symptom relief rather than disease modification. This is mainly due to the pathogenesis of the disease not yet being fully understood, resulting in a lack of a stepwise pharmacological approach to treatment. Drugs that are commonly used include SNRIs, tricyclic antidepressants (TCAs), cyclobenzaprine, pregabalin, gabapentin, acetaminophen, and nonsteroidal anti-inflammatory drugs (NSAIDs). Antidepressants and anticonvulsants are often utilized; however, treatment remains dependent upon the individual patient’s response.

Novel therapies, including cannabinoids, ketamine, lidocaine infusions, melatonin, low-dose naltrexone, and psychedelics, are supported by limited evidence, warranting further research. However, nonpharmacologic therapies such as CBT have been shown to improve pain by allowing patients to reach awareness and providing motivation to exercise as a means of reducing pain intensity. Studies have shown that consistent aerobic and resistance exercise are linked to improved pain tolerance and quality of life. In addition, holistic therapies such as cryotherapy and acupuncture may also offer benefits in certain patient populations.

In general, the current clinical practice guidelines for the management of fibromyalgia lack a stepwise approach due to the pathogenesis not yet being fully understood. Yet, treatment focuses mainly on symptom management, starting with non-pharmacological approaches including patient education, exercise, and CBT. If symptoms tend to persist, then pharmacological intervention is necessary with antidepressants and anticonvulsants most frequently utilized [11].

## Conclusions

Altogether, this case emphasizes the importance of keeping a high index of suspicion for fibromyalgia, especially when diagnostic tests produce unremarkable results. It is important for physicians to approach such patients with empathy and concern for their symptoms, as acknowledging their symptoms itself can play a therapeutic role. Further, this case stresses the value of a holistic approach to patient care. This includes evaluating the patient in a psychosocial context while keeping lifestyle factors and comorbidities in mind. As illustrated in this case, socioeconomic stressors have tremendous potential to exacerbate disease expression and treatment adherence, thus emphasizing the importance of individualized, patient-centered care. Additional research is still required to understand the pathogenesis of fibromyalgia to establish stepwise pharmacological treatment. Hence, this case underscores the necessity for clinicians to consider fibromyalgia early in patients with chronic unexplained pain, even in the presence of multiple comorbidities, and to integrate psychosocial factors into diagnostic reasoning.
